# Structure and photoluminescence properties of red-emitting apatite-type phosphor NaY_9_(SiO_4_)_6_O_2_:Sm^3+^ with excellent quantum efficiency and thermal stability for solid-state lighting

**DOI:** 10.1038/s41598-017-15595-z

**Published:** 2017-11-09

**Authors:** Lefu Mei, Haikun Liu, Libing Liao, Yuanyuan Zhang, R. Vasant Kumar

**Affiliations:** 1 Beijing Key Laboratory of Materials Utilization of Nonmetallic Minerals and Solid Wastes, National Laboratory of Mineral Materials, School of Materials Science and Technology, China University of Geosciences, Beijing, 100083 China; 2 Department of Materials Science and Metallurgy, University of Cambridge, Cambridge, CB3 0FS UK

## Abstract

A novel red-emitting phosphor NaY_9_(SiO_4_)_6_O_2_:Sm^3+^ (NYS:Sm^3+^) was synthesized and the X-ray diffraction and high-resolution TEM testified that the NYS compound belongs to the apatite structure which crystallized in a hexagonal unit cell with space group P6_3_/m. The novel phosphor boasts of such three advantageous properties as perfect compatible match with the commercial UV chips, 73.2% quantum efficiency and 90.9% thermal stability at 150 °C. Details are as follows. NYS:Sm^3+^ phosphor showed obvious absorption in the UV regions centered at 407 nm, which can be perfectly compatible with the commercial UV chips. The property investigations showed that NYS:Sm^3+^ phosphor emitted reddish emission with CIE coordination of (0.563, 0.417). The optimum quenching concentration of Sm^3+^ in NYS phosphor was about 10%mol, and the corresponding concentration quenching mechanism was verified to be the electric dipole–dipole interaction. Upon excitation at 407 nm, the composition-optimized NYS:0.10Sm^3+^ exhibited a high quantum efficiency of 73.2%, and its luminescence intensity at 150 °C decreased simply to 90.9% of the initial value at room temperature. All of the results indicated that NYS:Sm^3+^ is a promising candidate as a reddish-emitting UV convertible phosphor for application in white light emitting diodes (w-LEDs).

## Introduction

In the past decades, solid-state lighting technology has become commercially available with the advent of high performance *w*-LEDs, which represents a rapid evolution of new lighting systems designed to deliver improved illumination as well as new lighting features such as being environmentally friendly, offering long lifetime, enhancing energy-saving, and producing high luminous efficiency^1–5^. Presently, the commercial phosphor-converted *w*-LEDs use yellow-emitting (YAG:Ce^3+^) phosphor coated on a blue In-doped GaN chip or couple three red-, blue- and green-, (RGB tricolor) phosphors with near-ultraviolent (*n*-UV) chips^6–8^. However, this method faces problems of low color rendering index (CRI) and a high correlated color temperature (CCT) due to the deficiency of sufficient red spectral component. Therefore, it is necessary to explore alternative red or reddish phosphors which can be effectively excited in the UV range with excellent chemical stability^9–13^.

Selection of suitable host material is also an important factor for the preparation of luminescent materials for different applications. Compounds with oxy-apatite structure have been intensively studied owing to their excellent chemical and physical stabilities^14–17^. It is well known that oxy-apatite structure belongs to the hexagonal symmetrical system (space group of *P*6_3_/*m*) with a general chemical formula as A_10_[MO_4_]_6_O_2_, in which A is a cation (Na^+^, Ca^2+^, Y^3+^, La^3+^, etc.), while MO_4_ is an anionic group (PO_4_, SiO_4_, GeO_4_, etc.)^18–21^. As an important class of apatite-type silicate compounds, NaY_9_(SiO_4_)_6_O_2_ is isostructural with natural oxyapatite, which consists of two cationic sites: one is in a distorted 3-fold capped trigonal prism (*4 f* sites with *C*_3_ point symmetry), which is partially occupied by Y^3+^ (the site occupancy of 75%)−Na^+^ (the site occupancy of 25%) and nine oxygen ions, and the other is in a distorted pentagonal bipyramid (*6 h* sites with *C*_*s*_ point symmetry) coordinated with Y^3+^ and seven oxygen ions respectively. In 1982,Gunawardane first synthesized the apatite structure compound NaY_9_(SiO_4_)_6_O_2_, led its chemical composition and detailed crystal structure^22^. Later, Redhammer’s group found that the crystal structure of NaY_9_(SiO_4_)_6_O_2_ compound showed no symmetry change between room temperature and 100 K, and that the alterations in structural parameters were small^23^. Recently, apatite-type silicate compounds activated by rare earth have drawn great attention owing to their excellent luminescent characteristics^24–28^. You’s team reported the color-tunable phosphors of Ce^3+^/Tb^3+^/Eu^3+^/Mn^2+^ co-doped NaY_9_(SiO_4_)_6_O_2_^29^.

However, to our best knowledge, the luminescence properties of Sm^3+^ in NYS host have not yet been reported. As a member of efficient red-emitting trivalent rare earth ions, samarium (Sm^3+^) ions with sharp 4*f*-4*f* emission peak have been receiving intense research interests, widely used in luminescence materials because of their unique luminescent properties. Customarily, Sm^3+^ ions generate three dominant emissions located in reddish-emitting regions, which correspond to ^4^ *G*_*5/2*_ → ^6^ *H*_*5/2*_^4^, *G*_*5/2*_ → ^6^ *H*_*7/2*_ and ^4^ *G*_*5/2*_ → ^6^ *H*_*9/2*_ transitions respectively. However, the absorption efficiency of Sm^3+^ is low in the n-UV region where the 4*f*-4*f* electric dipole transitions are absolutely forbidden. Therefore, it is necessary to develop an excellent phosphor with good quantum efficiency to decrease the effect of the poor absorption efficiency.

In view that the effective ionic radii of Sm^3+^ ions is the closest to that of Y^3+^ ions, it may serve as an activator for reddish-emitting luminescence materials when Sm^3+^ ions are doped into the NYS matrix. Consequently, we demonstrate a red-emitting phosphor NYS:Sm^3+^ with apatite structure pumped for UV-light emitting diodes via a traditional solid-state method. Accordingly, the crystal structure of the NYS has been investigated. In addition, the concentration quenching as well as lifetime of Sm^3+^ in NYS phosphor was investigated. The excellent luminescence quantum efficiency and thermal stability indicate that as-prepared NYS:Sm^3+^ phosphor can act as a red UV convertible phosphor for *w*-LEDs.

## Experimental Section

The NYS:*x*Sm^3+^ (*x* = 0, 0.01, 0.05, 0.10, 0.15, 0.25, and 0.50) phosphors were prepared by a high temperature solid-state technique. The stoichiometric amounts of raw materials Na_2_CO_3_, SiO_2_ with analytic grade purity and Y_2_O_3_ (99.99%), Sm_2_O_3_ (99.99%) were weighed and mixed by grinding in an agate mortar; to compensate for loss of Na source at high temperature, a slight excess of Na_2_CO_3_ (5%wt beyond stoichiometry) was necessary, and the homogeneous mixture was transferred to an alumina crucible and annealed at 1400 °C for 4 h. The final products were cooled to room temperature and were reground for further measurements.

The structure of the final products was identified by using powder X-ray diffraction (XRD) analysis (XD-3, PGENERAL, China) in the 2θ range of 10° to 70°, with graphite monochromatized Cu Kα radiation (λ = 0.15406 nm) operating at 40 kV and 40 mA. The measurements of photoluminescence emission (PL) and photoluminescence excitation (PLE) spectra were performed by using a fluorescence spectrophotometer (F-4600, HITACHI, Japan) with a photomultiplier tube operating at 400 V, and a 150 W Xe lamp used as the excitation lamp. The room-temperature luminescence decay curves were obtained from a spectrofluorometer (Horiba, JobinYvon TBXPS) using a tunable pulse laser radiation as the excitation. Transmission electron microscope (TEM), high-resolution TEM (HRTEM), and selected area electron diffraction (SAED) analyses were checked by a JEOL JEM-2010 microscope with an accelerated voltage of 200 kV. The elemental analysis was carried out by energy dispersive spectroscopy (EDS) using an X-ray detector attached to the TEM instrument. The internal quantum efficiency was measured using the integrated sphere on the FLS920 fluorescence spectrophotometer (Edinburgh Instruments Ltd, UK), and a Xe900 lamp was used as an excitation source and white BaSO_4_ powder as a reference to measure the absorption. The signals were detected by a Hamamatsu R928P photomultiplier tube. The temperature-dependence luminescence properties were measured on the same spectrophotometer, which was combined with a self-made heating attachment and a computer-controlled electric furnace.

## Results and Discussion

**Figure 1:**
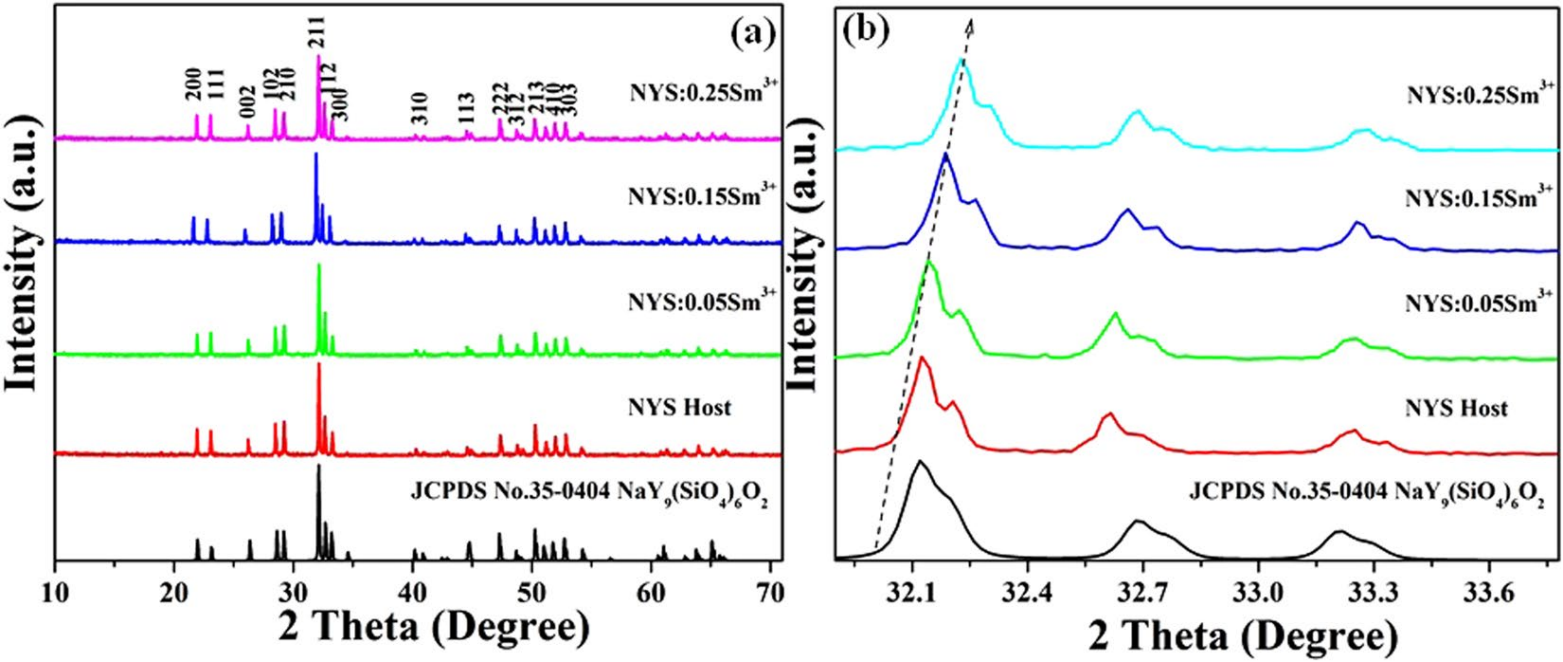
(**a**) XRD patterns of as-prepared NYS:*x*Sm^3+^ (*x* = 0, 0.05, 0.15 and 0.25). The standard data for NaY_9_(SiO_4_)_6_O_2_ (JCPDS card no. 35-0404) is shown as a reference. (**b**) XRD patterns of the enlarged profile in the range of 31.9 to 33.8°.

Figure 1(a) shows the XRD profiles of as-prepared NYS:*x*Sm^3+^(*x* = 0, 0.05, 0.15, and 0.25) samples, and Fig. 1(b) shows the enlarged spectrum profile in the range of 31.9 to 33.8°. It can be found that the sample spectra could be well indexed as pure crystalline NaY_9_(SiO_4_)_6_O_2_ phase (JCPDS no: 35–0404), which belongs to the hexagonal structure with the space group *P*6_3_/*m*, indicating that the introduction of Sm^3+^ ion into the NaY_9_(SiO_4_)_6_O_2_ lattice does not cause any significant change to the crystal structure of the host. Based on the effective ionic radii and charge balance of cations with different coordination numbers (CN), the activators Sm^3+^ ions are expected to occupy the Y^3+^ sites randomly in the NYS host, because the effective ionic radii of Sm^3+^ (*r* = 1.02 Å for CN = 7 and *r* = 1.132 Å for CN = 9) is the closest to that of Y^3+^ ions (*r* = 0.96 Å for CN = 7 and *r* = 1.075 Å for CN = 9)^30^. In addition, according to the enlarged profile, we can find that the diffraction peaks shift to a larger angle with *x* increasing, indicating the increase of the cell volume with the substitution of Sm for Y ions. This phenomenon also indicates that the doped Sm^3+^ are successfully incorporated in the host at Y^3+^ sites.

**Figure 2:**
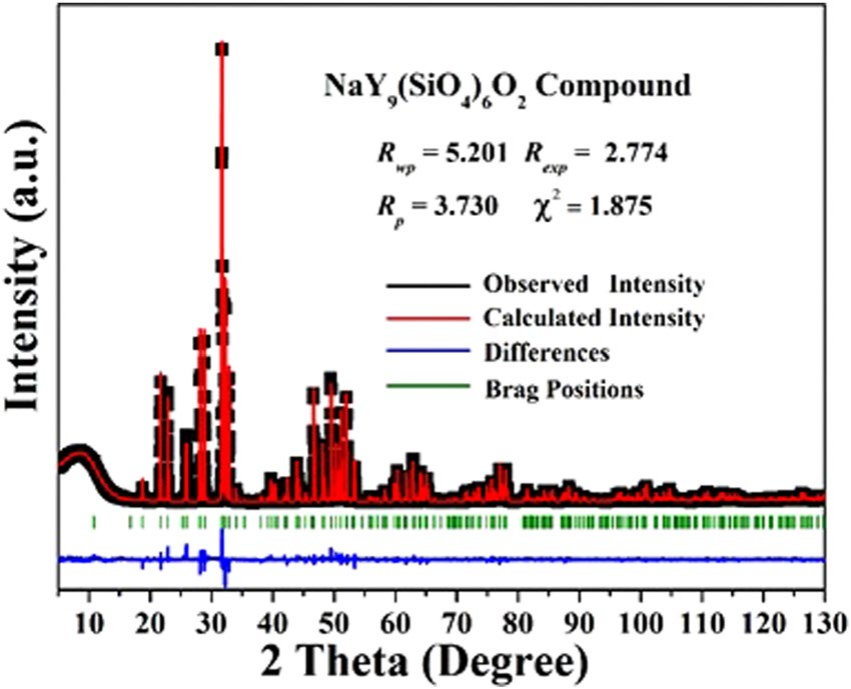
Rietveld refinement XRD patterns of NaY_9_(SiO_4_)_6_O_2_ at room temperature by TOPAS program. Solid red circles are calculated intensities, and black lines are the observed intensities. Short vertical lines show the position of Bragg reflections of the calculated pattern. Green solid lines below the profiles stand for the difference between the observed and the calculated intensities.

Figure 2 shows the observed (solid line), the calculated (red circles), and different (bottom) XRD profiles for the Rietveld refinement of NaY_9_(SiO_4_)_6_O_2_ with λ = 1.5406 Å by TOPAS program. Almost all peaks were indexed by hexagonal cell with parameters close to Cd_2_Nd_8_(SiO_4_)_6_O_2_ (apatite-type structure)^30^. Therefore crystal structure of Cd_2_Nd_8_(SiO_4_)_6_O_2_ was taken as starting model for Rietveld refinement. Sites of Cd/Nd ions were occupied by Na/Y ions and the occupation of sites was refined with assumption that their sum in each site equal to 1. Refinement was stable giving low R-factors (Table 1, Fig. 2).

**Table 1:** Fractional atomic coordinates and isotropic displacement parameters of NaY_9_(SiO_4_)_6_O_2_.

	*x*	*y*	*z*	*B*_iso_	Occ.
Y1	2/3	1/3	0.4912 (5)	0.8 (7)	0.75
Na1	2/3	1/3	0.4912 (5)	0.8 (7)	0.25
Y2	0.2325 (7)	0.2397 (2)	1/4	0.5 (7)	1
Si	0.4001 (7)	0.0274 (9)	1/4	1.0 (1)	1
O1	0.5962 (3)	0.4607 (2)	0.25	1.5 (2)	1
O2	0.3155 (2)	0.8275 (2)	0.25	1.5 (2)	1
O3	0.3390 (9)	0.0895 (8)	0.0678 (1)	1.5 (2)	1
O4	0	0	0.25	1.5 (2)	1

As mentioned previously, NYS belongs to apatite-type compound. In this compound, the Na(1)/Y(1) site is coordinated with nine O atoms: three O(1), three O(2) and three O(3), forming a Na/YO_9_-polyhedron with six short and three long Na/Y–O bonds, while the Y(2) site is coordinated by six oxygen atoms (O(1), O(2), four O(3), and one O(4) forming an irregular YO_7_-polyhedron. The Si atoms randomly occupy on the *6 h* site in the form of silicon-oxygen tetrahedron. The Na/YO_9_-polyhedron and YO_7_-polyhedron are connected by tetrahedral SiO_4_ groups and through sharing planes, edges and vertices^29^.

**Figure 3:**
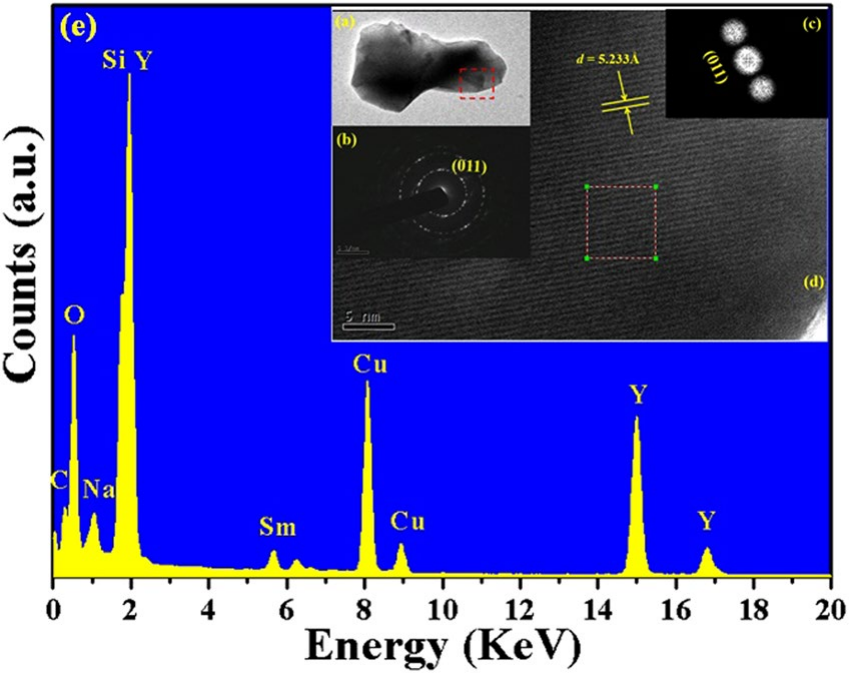
(**a**) TEM image, (**b**) SAED image, (**c**) FFTs image, (**d**) HRTEM image, (**e**) EDS spectrum of NaY_9_(SiO_4_)_6_O_2_:Sm^3+^ phosphor.

The fine local structures of NYS:Sm^3+^ were further examined by HRTEM. And Fig. 3 showed the five results of the examination taken from the selected micro-particle at the edge: (a) TEM image; (b) the selected area electron diffraction (SAED) image; (c) the fast Fourier transform (FFTs) image; (d) HRTEM image; (e) the energy dispersive spectroscopy (EDS) spectrum. It is found that the lattice fringes with a *d* spacing of 0.523 nm could be assigned to (011) plane. The *d*_(011)_ enlarged (*d*_(301)_ of 0.518 nm for NaY_9_(SiO_4_)_6_O_2_ compound)^22^ slightly due to the increase of the cell volume with the substitution of Sm for Y ions^31^. The SAED pattern of the as-prepared particle illustrates many diffraction spots in circles, indicating that the NYS:Sm^3+^ phosphor exhibits a highly crystalline nature with corresponding (011) reflections, which can be indexed to the hexagonal structure of NaY_9_(SiO_4_)_6_O_2_. In addition, energy dispersive spectroscopy (EDS) was performed to analyze the chemical composition of NYS:Sm^3+^ phosphor through which local information regarding concentration of various elements could be deduced. It confirmed the existence of the elements Na, Y, Si, O and Sm, with atomic ratios of 2.09:17.6:11.56:27.53:0.16, which was consistent with the result determined ratios of the starting materials.

**Figure 4:**
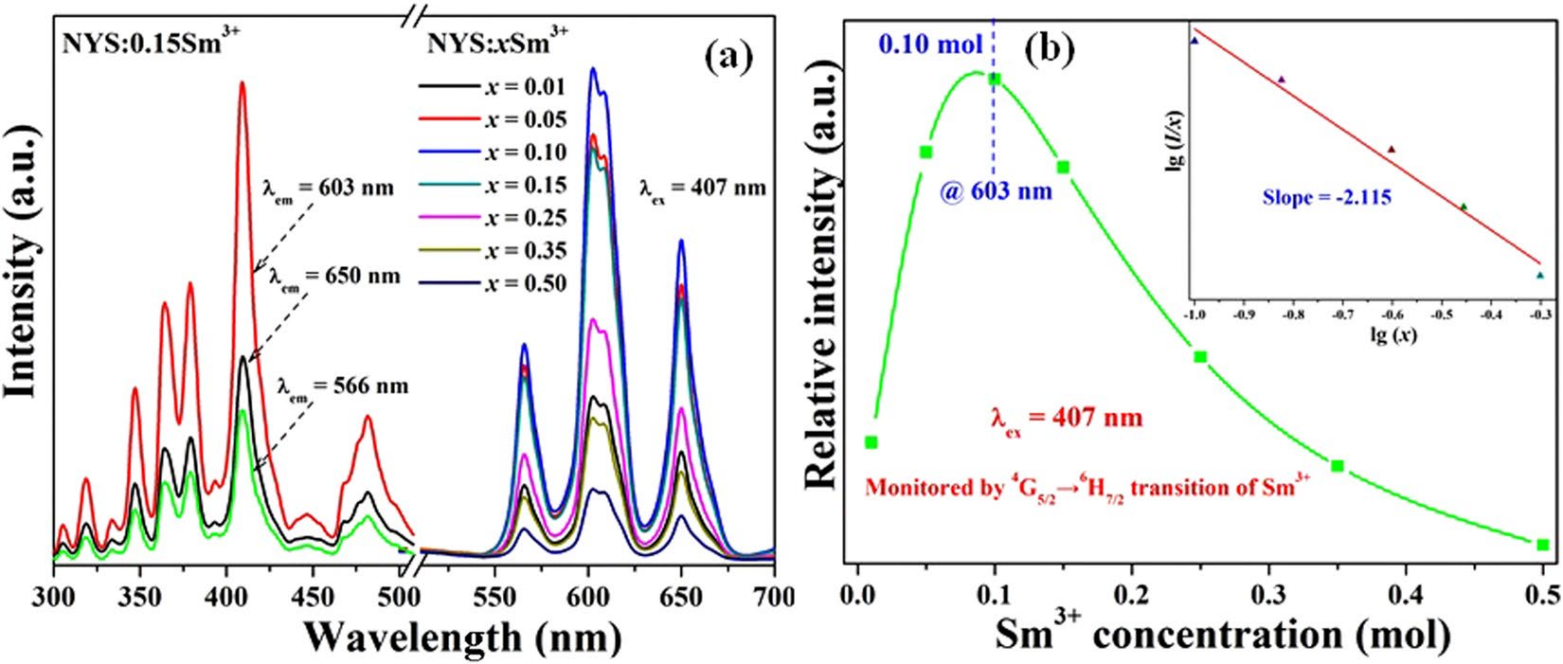
(**a**) Excitation (λem = 566, 603 and 650 nm) and emission spectra of NYS:*x*Sm^3+^ (*x* = 0.01 − 0.50) phosphors under 407 nm UV light excitation. (**b**) The dependence of the emission intensity on the concentration of Sm^3+^. The inset shows the fitting curves of lg(*I*/*x*) vs. lg(*x*) in NYS:*x*Sm^3+^ phosphors.

The photoluminescence excitation (PLE) spectra and PL spectra of NYS:*x*Sm^3+^ (*x* = 0, 0.01, 0.05, 0.15, 0.25, 0.35 and 0.50) under different monitoring wavelengths are demonstrated in Fig. 4a. It can be seen that the PLE spectrum of NYS:0.10Sm^3+^ monitored by 603 nm presents some observable band features ranging from 300 to 500 nm, which is in consistent with that monitored at 566 and 650 nm except for the tiny difference in the relative intensity. It is obvious that these sharp excitation peaks are located in the wavelength region of 300–500 nm appearing at 326, 345, 364, 378, 388, 407, 420, 441, 465, and 478 nm, which could be assigned to transitions from the ^6^H_5/2_ ground state to the excited states of Sm^3+4^, P_1/2_^4^, H_9/2_^4^, D_3/2_^4^, D_1/2_^4^, G_11/2_^4^, F_7/2_^4^, M_19/2_^4^, G_9/2_^4^, I_13/2_, and ^4^M_15/2_ levels, respectively^32,33^. In addition, the intense features absorption band at 300–500 nm suggests that this phosphor can be effectively excited by the UV LED chip. Under the excitation of 407 nm, the characteristic emission consists of three bands corresponding to the transitions from the ^4^G_5/2_ excited state to the ^6^H_5/2_ (566 nm), ^6^H_7/2_ (603 nm), and ^6^H_9/2_ (650 nm), respectively. Furthermore, the PL spectra of NYS:*x*Sm^3+^ (*x* = 0.01, 0.05, 0.10, 0.15, 0.25, 0.35 and 0.50) and the dependence of the integrated emission intensities on the concentration of Sm^3+^ ion under the excitation of 407 nm are also demonstrated in Fig. 4a,b. It is obvious that the emission intensity of Sm^3+^ ion first increases and reaches a maximum at *x* = 0.10, and then the emission intensity decreases as a result of concentration quenching effect. The concentration quenching may be induced by cross relaxation processes due to proximity in Sm^3+^ ions. With increasing the Sm^3+^ ions doping concentration, there is more possibility of enhancing the energy transfer within Sm^3+^ ions beyond the optimal doping concentration through non-radiative process^34^. If we consider energy transfer between two identical centers, the critical distance (*Rc*) is defined as the distance for which the probability of energy transfer equals the probability of radiative emission of Sm^3+^, as pointed out by Blasse^35^. The average separation *R*_*c*_ can be estimated according to the following equation^36–38^:

1 $${R}_{c}\approx 2{(\frac{3V}{4\pi {x}_{c}N})}^{\frac{1}{3}}$$

where *V* is the volume of the unit cell, *x*_*c*_ is the critical concentration, and *N* is the number of available sites for the dopant in the unit cell. In our host of NYS, *N* equals to 1, and *V* is estimated to be 508.98 Å^3^, and the critical doping content *x*_*c*_ = 0.10. According the Equation (1), the critical distance was determined to be about 21.34 Å. It is of little possibility for energy transfer through the exchange interaction mechanism with longer distance. Thus, the electric multipolar interaction will take place for energy transfer between two Sm^3+^ ions. In order to further describe internal concentration quenching effect, interaction between sensitizers or between a sensitizer and an activator can be expressed by the following equation^39–41^:

2 $$\frac{I}{x}=K{[1+\beta {(x)}^{\frac{\theta }{3}}]}^{-1}$$

where *x* is the activator concentration which is higher than the critical concentration, *I*/*x* is the emission intensity (*I*) per activator concentration (*x*), *K* and *β* are constants for the same excitation condition, and *θ* is an indication of electric multipolar character, *θ = *6, 8, 10 for dipole-dipole (*d-d*), dipole-quadrupole (*d–q*), or quadrupole-quadrupole (*q–q*), respectively^42^. The insert of Fig. 4b shows the fitting lines of *log*(*I/x*) vs *log*(*x*) in NYS:*x*Sm^3+^ phosphors at 603 nm beyond the quenching concentration. It is clear that the fitting curves of *lg*(*I/x*) vs *lg*(*x*) can be well matched as linear dependence. The slope of the straight line was determined to be −2.115. Herein, the value of *θ* can be calculated as 6.345, which is close to 6 consistent with the quenching results arising from dipole-dipole interactions in the NYS:*x*Sm^3+^ phosphor.

**Figure 5:**
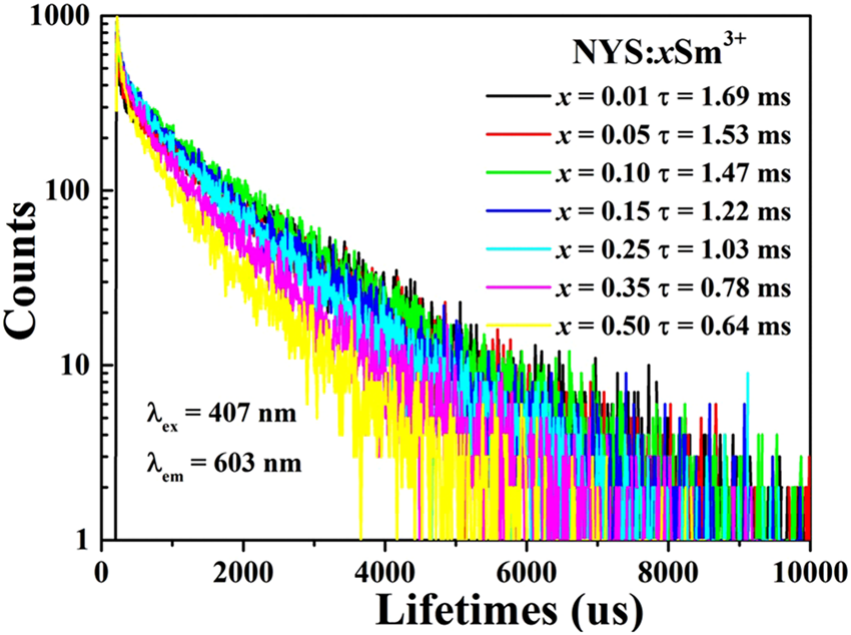
Decay curves of Sm^3+^ emission in NYS:*x*Sm^3+^ (*x* = 0.01−0.50) phosphors under excitation at 407 nm, monitored at 603 nm at room temperature.

The decay curve of Sm^3+^ emission for NYS:*x*Sm^3+^ (*x* = 0.01, 0.05, 0.10, 0.15, 0.25, 0.35 and 0.50) excited at 407 nm was measured as shown in Fig. 5. It can be found that all of the decay curve can be fitted successfully, based on a typical second-order exponential decay equation as follows^43^:

3 $$I(t)={I}_{0}+{A}_{1}\exp (-t/{\tau }_{1})+{A}_{2}\exp (-t/{\tau }_{2})$$

where *I* is the luminescence intensity at time *t* and *I*_0_ are the luminescence intensity initially, *A*_1_ and *A*_2_ are constants,*τ*_1_ and *τ*_2_ are the lifetimes for the exponential components. Furthermore, the effective lifetime constant (*τ* ^*^) can be calculated as:

4 $${\tau }^{\ast }=({A}_{1}{{\tau }_{1}}^{2}+{A}_{2}{{\tau }_{2}}^{2})/({A}_{1}{\tau }_{1}+{A}_{2}{\tau }_{2})$$

The average decay times (*τ* ^*^) were calculated to be 1.69, 1.53, 1.47, 1.22, 1.03, 0.78 and 0.64 ms for NYS:*x*Sm^3+^ with *x* = 0.01, 0.05, 0.10, 0.15, 0.25, 0.35 and 0.50, respectively. An obvious phenomenon is obtained that the decay time begins to decrease gradually with Sm^3+^ concentration increasing. Considering the distance between Sm^3+^-Sm^3+^ ions decreases in pace with increasing Sm^3+^ doped concentrations, the probability of energy transfer to luminescent killer sites increased, thereby the lifetimes of Sm^3+^ ions are shortened due to favorable non-radiative energy transfer processes as Sm^3+^ concentration increases, which is similar to some previous results discussed in other systems^44–46^.

**Figure 6:**
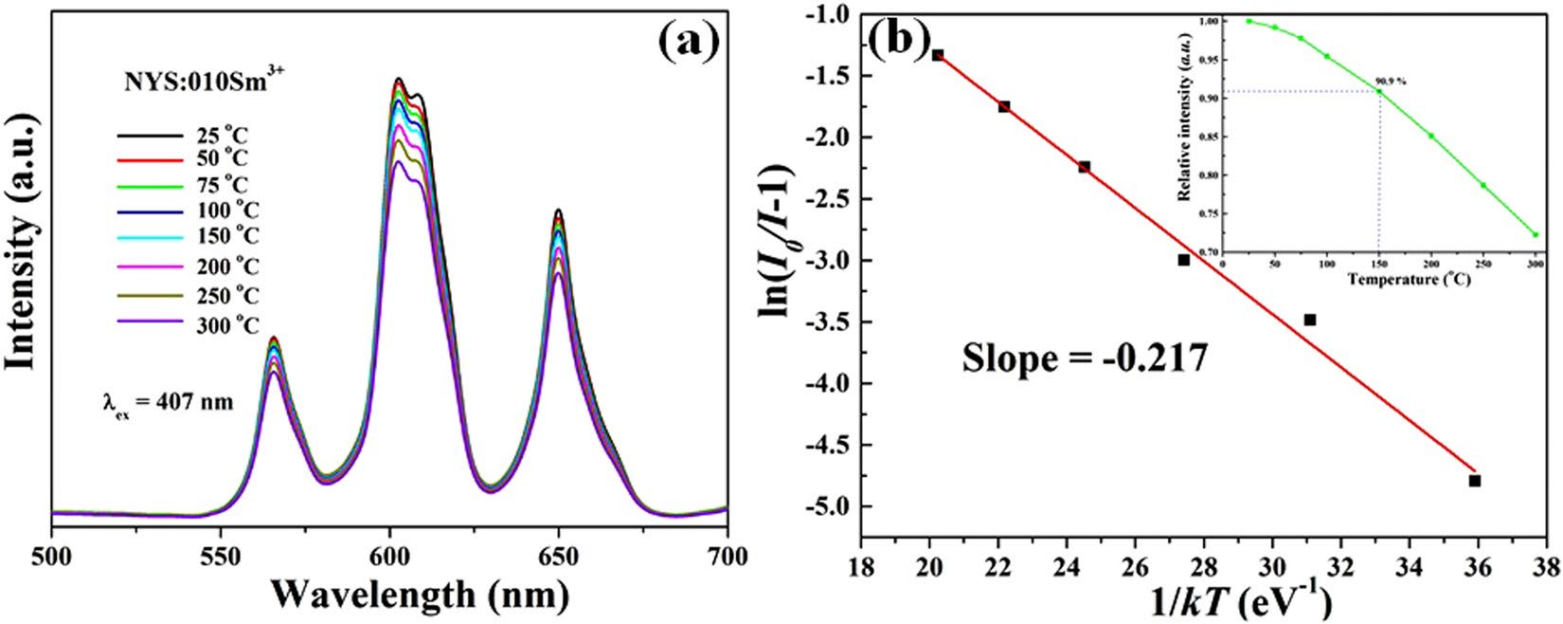
(**a**) Temperature-dependent PL spectra of NYS:0.10Sm^3+^ under different temperatures in the range of 25–300 °C. (**b**) A ln[(I_0_/I_T_)^−1^] vs 1/kT activation energy graph for thermal quenching of NYS:0.10Sm^3+^ phosphor.

In order to further investigate the possible practical application in high power *w*-LEDs of the NYS:Sm^3+^ phosphor, the temperature dependent emission spectra of NYS:0.10Sm^3+^phosphor ranging from 25 to 300 °C monitored by 407 nm is shown in Fig. 6a. It depicts that the position and shape of the emission spectra do not change with increasing temperatures. While the temperature increases, the intensity of the emission spectrum decreases. As given in the inset of Fig. 6b, when the temperature turns up to 150 °C, the emission intensity is 90.9 percent of that at 25 °C, which indicates this phosphor has excellent thermal stability for potential application in *w*-LEDs. In order to understand the temperature dependence behavior better, an Arrhenius fitting of the emission intensity of NYS:0.10Sm^3+^ phosphor and the calculated activation energy (Δ*E*) for thermal quenching were carried out and the results were given in Fig. 6b. The activation energy (Δ*E*) can be expressed by Equation (5)^26,47^:

5 $${I}_{T}=\frac{{I}_{0}}{1+c\exp (-\frac{{\rm{\Delta }}E}{kT})}$$

where *I*_0_ is the initial emission intensity of the phosphor at room temperature, *I*_T_ is the emission intensity at different temperatures, *c* is a constant, *ΔE* is the activation energy for thermal quenching, and *k* is the Boltzmann constant (1.38 × 10^−23^ J/K). As shown in the inset, the plot of ln[(*I*_0_/*I*_T_) − 1] vs. 1/*k*T yields a straight line, and the corresponding activation energy Δ*E* is obtained 0.217 eV.

**Figure 7:**
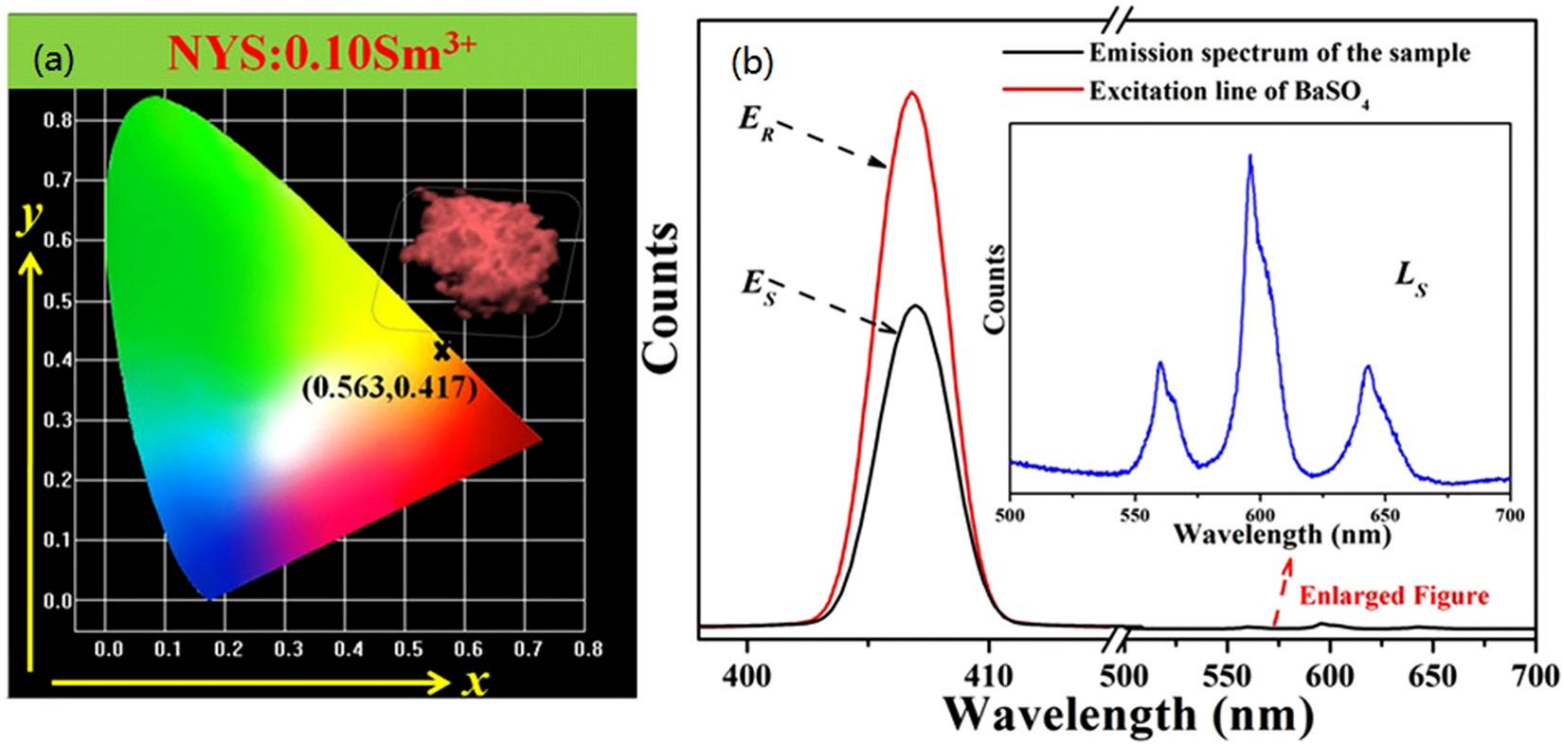
(**a**) CIE chromaticity diagram for NYS:0.10Sm^3+^ phosphor excited at 407 nm and the digital photos of the samples under 365 nm UV lamp excitation. (**b**) Excitation line of BaSO_4_ and emission spectrum of NYS:0.10Sm^3+^ phosphor collected by using an integrating sphere. The inset shows a magnification of the emission spectrum.

The chromaticity diagram and CIE coordinates are very important to disclose the exact emission color and color purity of the sample. The CIE chromaticity diagram for the NYS:0.10Sm^3+^ phosphor under 365 nm UV excitation is shown in Fig. 7a. It can be seen that the emission color of the as-prepared samples located in the reddish light region. The calculated color coordinate is found to be (0.563, 0.417), which indicates the phosphor can be used as a reddish-emitting candidate phosphor for *w*-LEDs application as the obtained CIE chromaticity coordinate is very much close to the Nichia corporation developed amber LED NSPAR 70BS (0.575, 0.425)^48^. A digital photo of the NYS:0.10Sm^3+^ phosphor under 365 nm UV lamp is shown in the inset of Fig. 7a revealing an intense reddish light. Additionally, we have also measured the internal quantum efficiency (QE) of NYS:0.10Sm^3+^ phosphor according to the reported method. On the basis of the result of Fig. 7b, the internal QE value can be calculated by following formula^49,50^:

6 $${\eta }_{QE}=\frac{\int {L}_{S}}{\int {E}_{R}-\int {E}_{S}}$$

where *L*_*S*_ is the luminescence emission spectrum of the sample; *E*_*S*_ is the spectrum of the light used for exciting the sample; *E*_*R*_ is the spectrum of the excitation light without the sample in the sphere. The measured internal QE of NYS:0.10Sm^3+^ phosphor is determined as 73.2% under 407 nm excitation. As a reference, we have also measured the QE value of the commercial red phosphor Y_2_O_2_S:Eu^3+^, and the value is 80.9% under 393 nm excitation. It is believed that as-prepared NYS:0.10Sm^3+^ phosphor can act as a potential reddish-emitting UV convertible phosphor for *w*-LEDs with high luminescence quantum efficiency.

## Conclusion

In summary, a red-emitting apatite-type phosphor NaY_9_(SiO_4_)_6_O_2_:Sm^3+^ has been synthesized and its properties have been reported in this paper. The phase purity and fine local structure were examined by XRD and HRTEM. This phosphor showed strong absorption in the UV regions and the optimum excitation band was located at 407 nm region, which matched well with the commercial UV chips (350–410 nm). The emission spectrum demonstrated that the characteristic emission consists of three bands, which corresponded respectively to the transitions from the ^4^G_5/2_ excited state to the ^6^H_5/2_ (566 nm)^6^, H_7/2_ (603 nm), and ^6^H_9/2_ (650 nm). The critical Sm^3+^ quenching concentration (QC) was estimated to be about 10%mol, and the corresponding QC mechanism was verified to be based upon dipole–dipole interactions. The fluorescence decay curves, temperature dependence photoluminescence and CIE value of NYS:Sm^3+^ phosphors have been discussed in detail. The excellent thermal stability and internal quantum efficiency indicated that the as-prepared NYS: Sm^3+^ phosphors are potential red-emitting UV convertible phosphor for applications in the next generation w-LEDs.
